# Minimally invasive palliative treatment of malignant tracheoesophageal fistula using cardiac septal occluder

**DOI:** 10.1007/s00423-024-03363-3

**Published:** 2024-06-01

**Authors:** Lin Teng, Fei Zhou, Xiaoqi Xiong, Haoyu Zhang, Linchen Qiao, Zaiqiang Zhang, Qin Qin, Xinyu Song

**Affiliations:** 1 Department of Cardiology, Yichang Central People’s Hospital, The First College of Clinical Medical Sciences, China Three Gorges University, Yichang, 443003 Hubei People’s Republic of China; 2 Department of Respiratory, Yichang Central People’s Hospital, The First College of Clinical Medical Sciences, China Three Gorges University, NO,183 Yiling Road, Yichang, 443003 Hubei People’s Republic of China

**Keywords:** mTEF, Cardiac septal occluder, Closure, Minimally invasive, Treatment, Complications

## Abstract

**Introduction:**

Tracheoesophageal fistula (TEF) especially malignant TEF (mTEF) is an uncommon yet critical medical condition necessitating immediate intervention. This life-threatening condition frequently manifests in critically ill patients who are dependent on prolonged mechanical ventilation and are unsuitable candidates for thoracotomy due to their compromised health status. The Management of these mTEF patients remain a significant challenge.This study aimed to evaluate the safety and efficacy of using a cardiac septal occluder for the closure of mTEF.

**Methods:**

8 patients with mTEF underwent closure surgery using atrial/ventricular septal defect (ASD/VSD) septal occluders at the Respiratory Department of HuBei Yichang Central People’s Hospital from 2021 to 2023. The procedure involved percutaneous placement of the occluder through the fistula to achieve closure.

**Results:**

The placement of the cardiac septal occluder was successfully achieved with ease and efficiency in all patients. The study demonstrated that the use of cardiac septal occluder therapy in patients with mTEF can alleviate symptoms, improve quality of life, and enhance survival rates, with no significant complications observed. Furthermore, the study provided comprehensive details on surgical indications, preoperative evaluation and diagnosis, selection of occluder, methods of occlusion, and postoperative care.

**Conclusions:**

The application of cardiac septal occluder in the treatment of mTEF is a safe and effective palliative treatment. This approach may be particularly beneficial for patients with a high risk of complications and mortality associated with traditional surgical interventions.

**Supplementary Information:**

The online version contains supplementary material available at 10.1007/s00423-024-03363-3.

## Introduction

Malignant tracheoesophageal fistula (mTEF) is an aberrant pathological channel formed between the esophagus and trachea due to direct infiltration of malignant tumors or necrosis and perforation of the esophageal or tracheal walls during the course of primary tumor treatment [1]. mTEF commonly occurs as an advanced complication of malignant tumors, with a reported incidence rate of 5–15% in the past two decades [2]. Its main clinical manifestations include choking during swallowing, recurrent pulmonary infections, chronic cough with sputum production, respiratory distress, and weight loss [3]. Without appropriate intervention, mTEF can lead to mortality, with a median survival period ranging from 6 to 12 weeks [4]. The key to improving survival lies in the successful closure of the fistula, which is crucial for managing the two major life-threatening complications: pulmonary infection and malnutrition. Surgical treatment may not be suitable for patients with compromised health conditions, necessitating a personalized approach based on individual patient characteristics [4, 5].

Traditional surgical approaches for TEF closure, such as thoracotomy, have been associated with high mortality, particularly in elderly or frail patients due to their increased susceptibility to complications and prolonged hospital stays [6, 7]. However, minimally invasive techniques have emerged as alternative options for mTEF management, including endoscopic closure, stent placement, and laser-assisted repair etc. Nonetheless, the success rates of these minimally invasive techniques vary and depend on the size and location of the mTEF, as well as the overall health status of the patient [8]. The effectiveness and safety of these techniques are still being investigated, and the long-term outcomes are yet to be fully elucidated.

Currently, the application of cardiac septal occluder for the closure of TEF has been the subject of investigation in several studies, which have demonstrated promising outcomes in terms of safety and efficacy. This technique involves the percutaneous placement of a cardiac septal occluder to achieve closure of the fistula, thereby avoiding the need for extensive surgical dissection [9–12]. However, it should be noted that most of these studies lack standardized guidelines for the procedure, relying mainly on individual experiences. The feasibility of occlusion techniques for TEF has not been fully explored and requires further investigation [10]. Moreover, currently there are relatively few reported cases regarding the application of a cardiac septal occluder for the treatment of mTEF. In this study, we conducted a retrospective analysis of 8 cases involving patients with mTEF who underwent closure surgery using atrial/ventricular septal defect (ASD/VSD) septal occluder at the Respiratory Department of HuBei Yichang Central People’s Hospital from 2021 to 2023. Our findings demonstrated that the utilization of a cardiac septal occluder is a safe and effective approach for mTEF closure, with no significant complications observed. Additionally, we comprehensively summarized the closure surgical indications, preoperative evaluation and diagnosis, surgical instruments and materials, selection of occluder, methods of occlusion, and postoperative care. These findings provide valuable guidance for the clinical application of cardiac septal defect occluder in the management of TEF.

## Materials and methods

### Materials

**Fig. 1:**
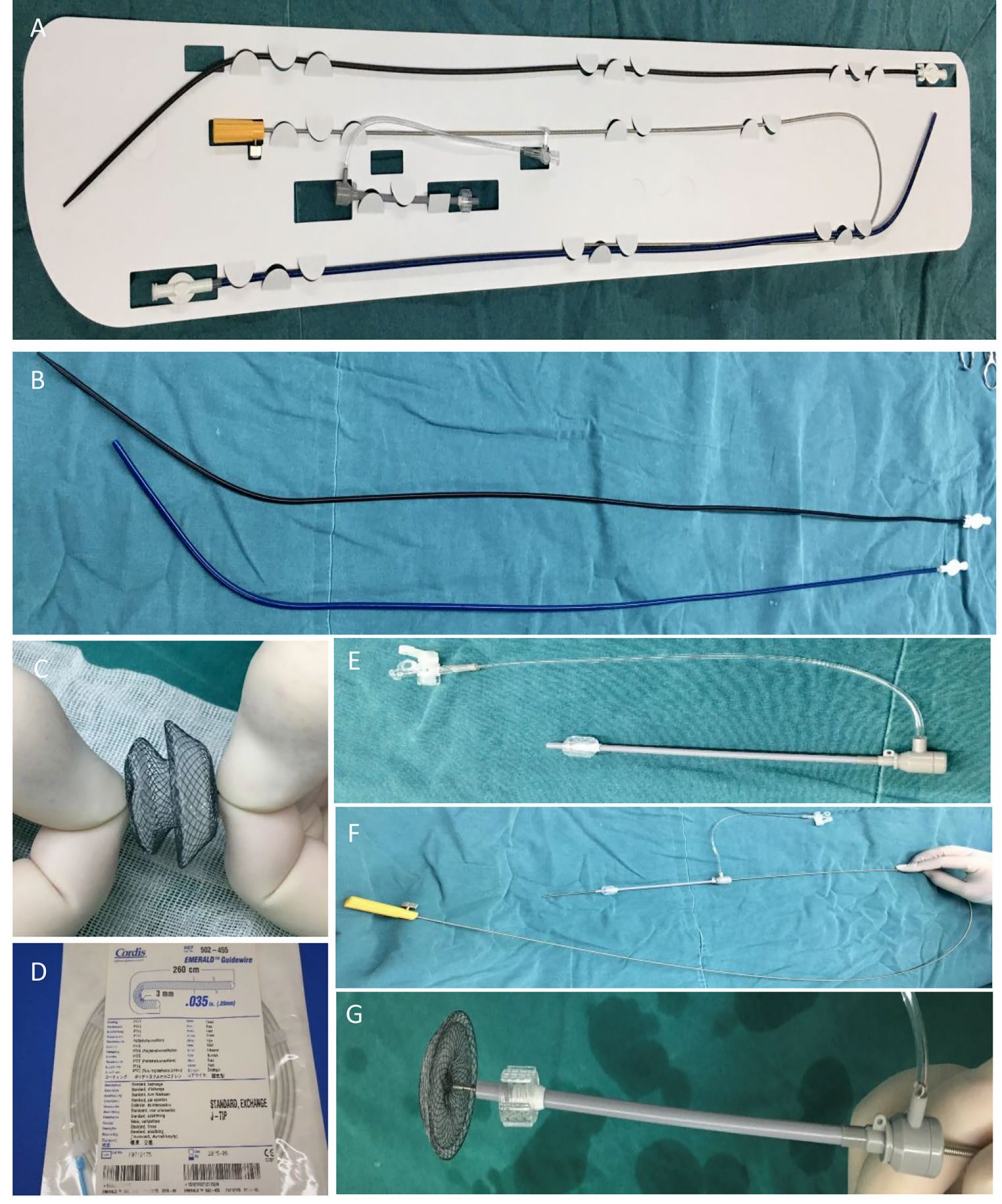
Materials of treating TEF with cardiac septal occlude. **(A)** Delivery system; **(B)** Delivery sheath; **(C)** Cardiac septal occlude; **(D)** 0.35” x 260 cm J-tip emerald guidewire; **(E)** Loading Device; **(F)** Delivery cable; **(G)** Preloading of cardiac septal occluder onto Delivery Cable

The occlusion procedure involved the utilization of various materials and equipment, including a cardiac ASD/VSD septal occluder, a delivery sheath, a loading device, and a delivery cable (Chinese Medicine Saint Jie, Beijing, China), a 0.35” x 260 cm J-tip Emerald guidewire (Cordis 502455 Standard Tip), a rigid bronchoscope, a fiberoptic bronchoscope, an electrocardiography monitor, and a ventilator, among others [Fig. 1]. The selection of an appropriate occluder for TEF closure lacks standardized guidelines. The choice of the occluder size for each individual case should be carefully evaluated based on the location, size, and characteristics of the TEF.

### Methods : *procedure and technique*

After obtaining approval from the Ethics Committee of Yichang Central People’s Hospital (Approval No. 2021-026-01), we conducted a retrospective analysis of data from eight patients with advanced mTEF who received treatment at the Respiratory Department of our hospital from 2021 to 2023. The patients included in the study underwent placement of a cardiac septal occluder under general anesthesia using a fiberoptic bronchoscope, with direct visualization for fistula occlusion. Written consent was obtained from all patients for both the research and publication purposes.

**Fig. 2:**
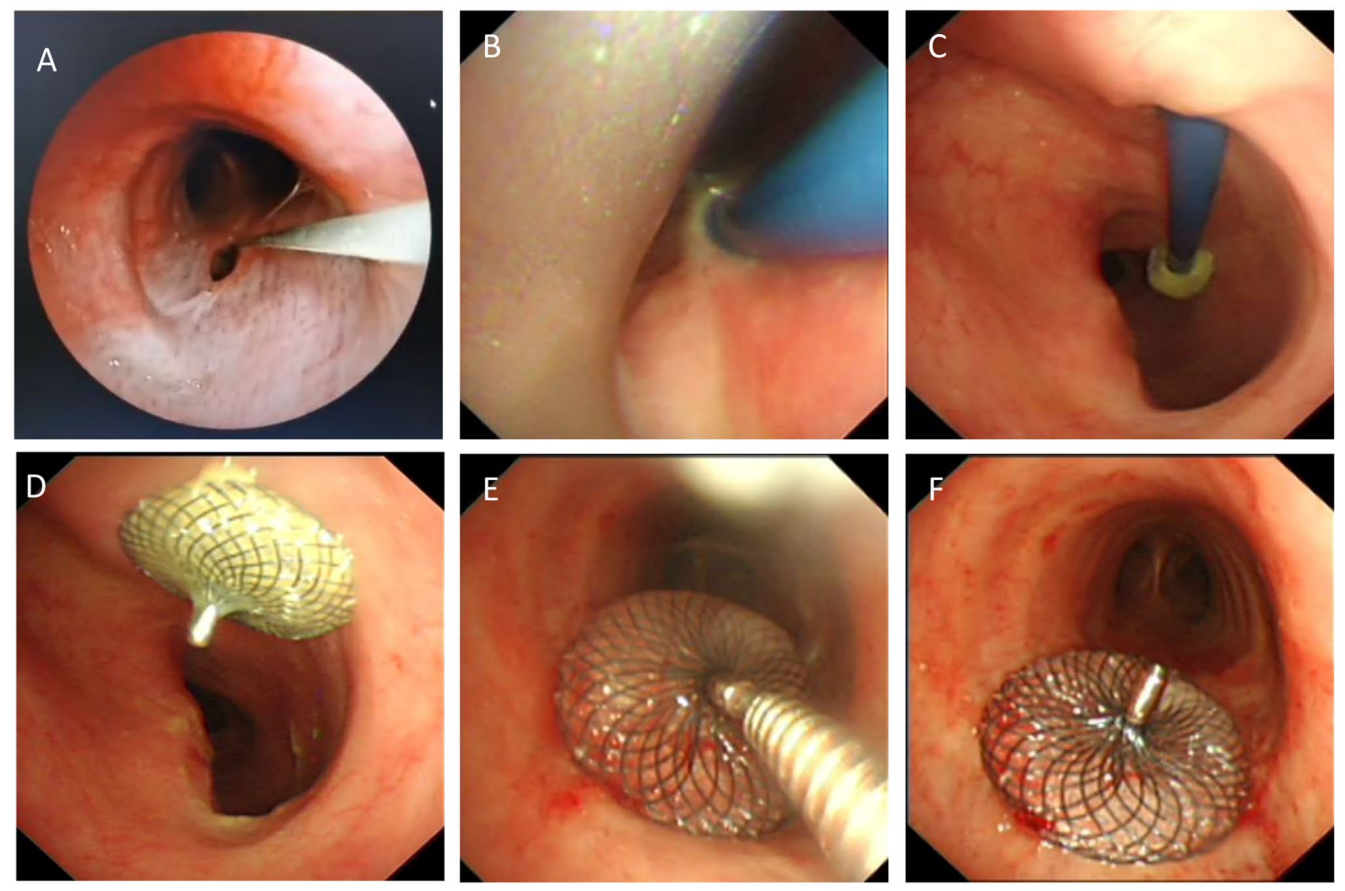
Surgical procedure of treating mTEF with cardiac septal occlude. **(A)** Guide wire passes through bronchial fistula into esophagus; **(B)** Placement of delivery sheath; **(C)** Introducing cardiac septal occluder through fistula opening to esophagus via delivery sheath; **(D)** Positioning of distal end of occluder against the esophageal wall; **(E)** Counterclockwise rotation detaches cable from occluder; **(F)** Positioning of proximal end of occluder against the tracheal wall

All the closure surgical procedures were performed under general anesthesia with deep sedation and analgesia, with continuous monitoring of the patient’s vital signs and blood oxygen saturation using electrocardiography. Initially, a rigid bronchoscope or laryngeal mask was inserted orally and connected to the ventilator for assisted respiration. Subsequently, the bronchoscope was used to confirm the location and size of the TEF opening on both the tracheal and esophageal sides. Under bronchoscopic guidance, a guidewire was passed through the bronchoscope forceps channel into the trachea, and then passed through the fistula into the esophagus[Figure 2A]. After removing the bronchoscope, the guide wire is used to guide the 45° ASD delivery sheath into the trachea, with the distal end passing through the fistula and into the esophagus to establish the surgical pathway (The size of the delivery sheath was chosen according to the actual size of the fistula) [Fig. 2B]. Next, the appropriate size cardiac septal occluder was preloaded onto a delivery cable and connected to it by rotating it clockwise using a screw connection system. The occluder was then sent into the esophagus through a delivery sheath [Fig. 2C]. The delivery sheath was then withdrawn, and the occluder was deployed under direct visualization through the esophagus. The occluder was gently pulled to fit snugly against the esophageal wall [Fig. 2D]. Subsequently, the bronchoscope was reintroduced into the trachea to slowly withdraw the delivery sheath under direct visualization and release the proximal occluder on the tracheal side [Fig. 2E]. The occluder was then gently pushed and pulled with the cable to ensure that it was fixed at the tracheoesophageal fistula, completely occluding the fistula and ensuring unobstructed passage of the trachea and esophagus. Finally, the cable was then rotated counterclockwise to detach it from the occluder, and both the cable and sheath were withdrawn to complete the surgery [Fig. 2F]. The entire surgical procedure takes approximately 30 to 60 min (Video 1).

## Results

### Clinical presentation

The study cohort comprised 8 patients with mTEF, with a median age of 72.9 years (range, 65–80 years) and a median body weight of 55.6 kg (range, 44–69 kg), including 6 males and 2 females. Among them, 5 patients (62.5%) were diagnosed with esophageal cancer, and 3 patients (37.5%) had lung cancer. All patients had a history of smoking and/or alcohol consumption. No other significant medical histories or surgical contraindications were noted. Hospital admission for these patients was primarily due to symptoms such as aspiration, coughing, inability to eat, and severe respiratory infections leading to sepsis.

**Fig. 3:**
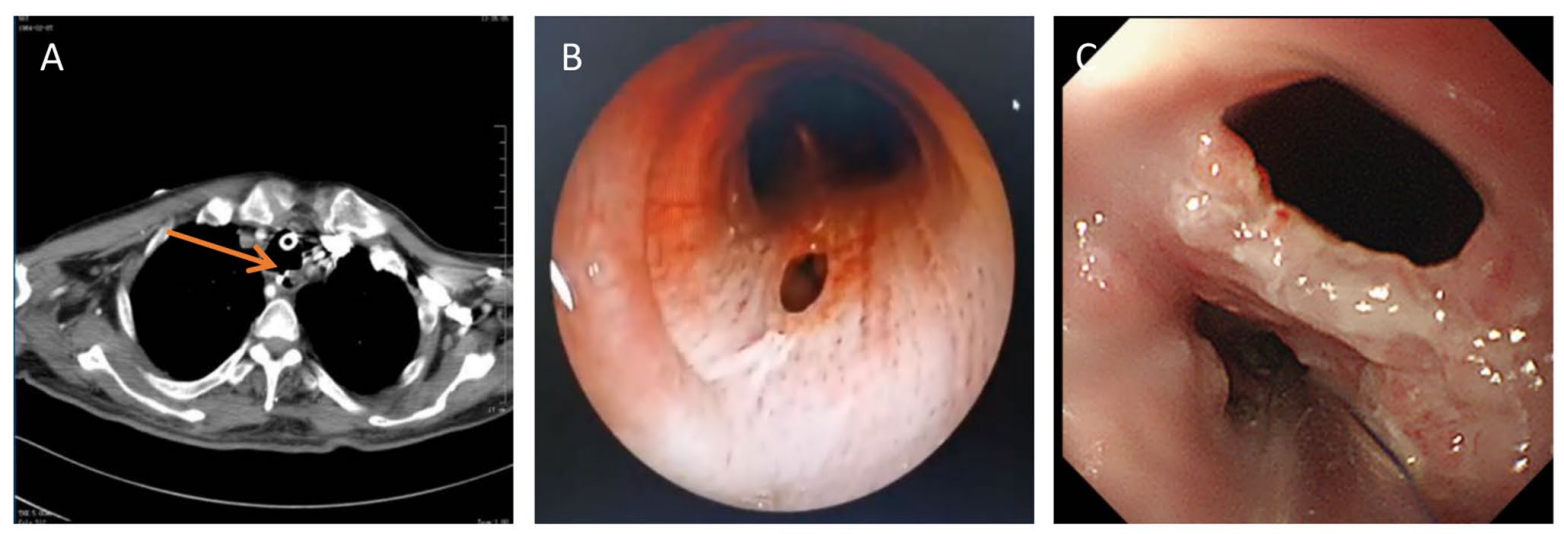
Diagnosis of TEF. **(A)** Diagnosis of TEF with chest CT ; **(B)** Confirmation of TEF location and size with bronchoscopy on tracheal side; **(C)** Confirmation of TEF location, size and cause with Digestive endoscopy on esophageal side

All patients diagnosed with mTEF underwent a comprehensive preoperative diagnostic procedure, which included chest CT scan [Fig. 3A], bronchoscopy examination [Fig. 3B], and digestive endoscopy [Fig. 3C]. The mean size of the fistula opening was 8.6 mm (range, 6–14 mm). Surgical intervention for occlusion was indicated for mTEF cases with a diameter larger than 3 mm, assuming no significant tracheal lumen stenosis was present. Patients with mTEF who had the following contraindications were excluded from the study: severe cardiopulmonary insufficiency that would hinder the tolerability of minimally invasive bronchoscopy examination, uremia, recent myocardial infarction within one-month, cerebrovascular accidents, severe epilepsy, coronary heart disease, acute and chronic renal failure, severe hypertension, etc(Table 1). In our study, among the 8 patients who underwent successful closure of mTEF, 2 cases (25%) opted for ASD closure devices, while 6 cases (75%) chose VSD closure devices. The selection of the occluder size can be based on sizes commonly used for treating congenital heart disease, with the occluder size after expansion being greater than 3–4 mm.

**Table 1:** Patient characteristics at the time of surgery

Patient number	gender	Age(years)	Weight(kg)	Etiology	Sepsis	Fistula size (mm)	Nasal Feeding	Tracheal stenosis	smoking and/or alcohol
1	Male	72	56	esophageal cancer	Yes	6	Yes	NO	smoking/alcohol
2	Female	80	44	lung cancer	Yes	7	Yes	NO	alcohol
3	Male	76	69	esophageal cancer	Yes	12	Yes	NO	smoking/alcohol
4	Male	65	53	lung cancer	Yes	8	Yes	NO	smoking
5	Male	77	55	esophageal cancer	Yes	6	Yes	NO	smoking
6	Male	69	62	esophageal cancer	Yes	7	Yes	NO	smoking
7	Female	74	49	lung cancer	Yes	9	Yes	NO	alcohol
8	Male	70	58	esophageal cancer	Yes	10	Yes	NO	smoking/alcohol

### Postoperative care and follow-up

**Fig. 4:**
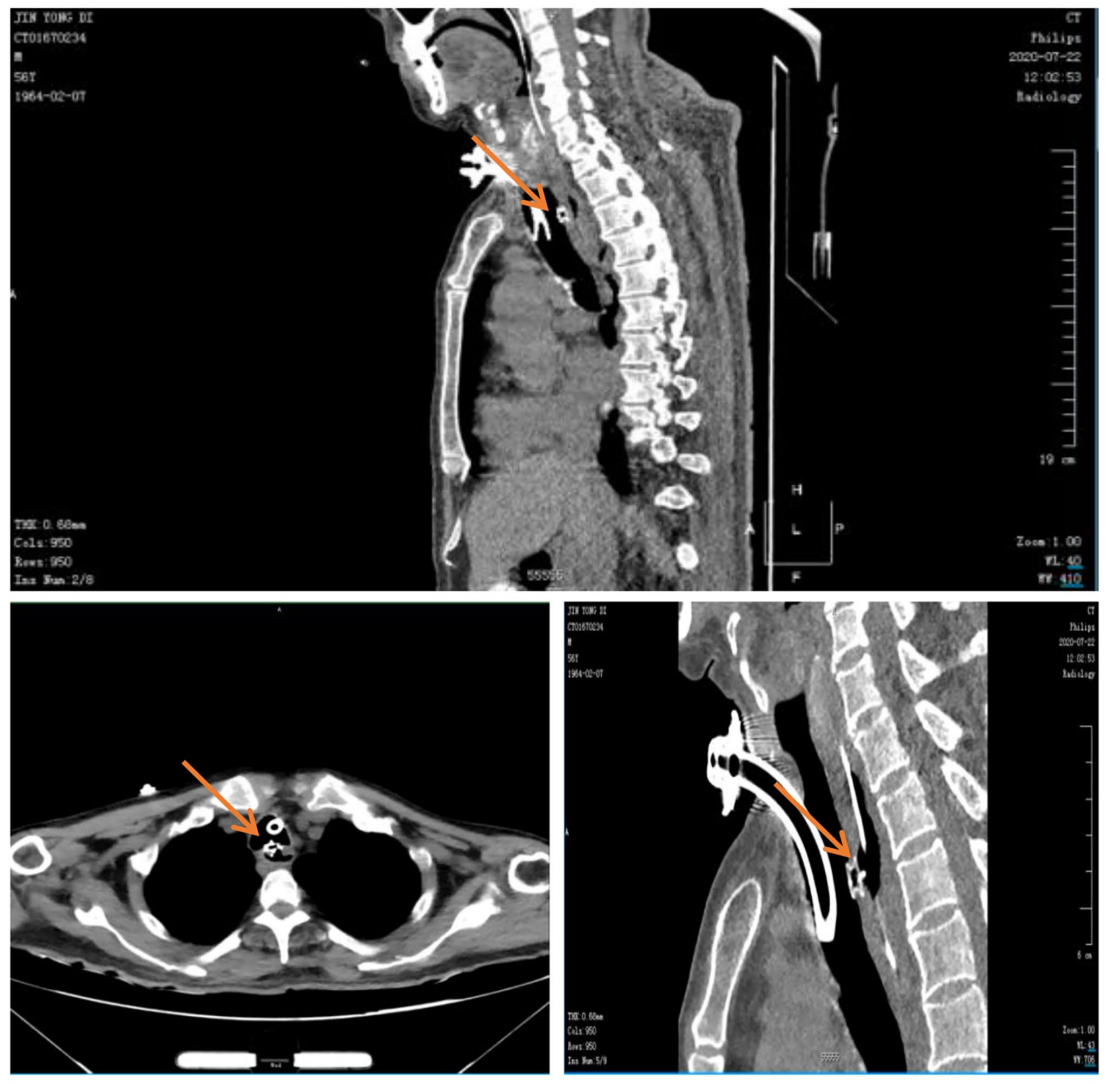
Transverse and sagittal views of occluder position on postoperative CT. The location of the occlusion device is indicated by the arrow

Following mTEF occlusion, all patients should undergo close monitoring in the respiratory intensive care unit (RICU). Assisted ventilation may be required for 1 to 2days, and vigilant surveillance for signs of infection or bleeding is crucial. Antibiotics and other medications may be administered to prevent and treat infections, while analgesics can be given for effective pain management. Postoperative chest CT should be performed to ensure optimal placement of the occluder [Fig. 4]. Following the procedure, enteral feeding was initiated through a nasal feeding tube and continued for a duration of 15 to 30 days. The removal of the nasal feeding tube was considered after follow-up evaluation confirmed the secure fixation of the occlusion device with epithelialization and absence of leakage on esophagography. Once the nasal feeding tube was removed, the patient was able to resume oral intake. Patients may require extended hospital observation prior to discharge, and regular follow-up is essential to detect any potential complications, especially respiratory distress or airway obstruction. In the event of severe coughing, respiratory distress, or decreased blood oxygen saturation postoperatively, emergency bronchoscopy should be promptly performed to assess if the occlusion device has dislodged, potentially causing airway obstruction. If the detachment of the occluder is confirmed, immediate removal of the device should be performed under general anesthesia using methods such as forceps and mesh baskets via rigid bronchoscopy.

**Fig. 5:**
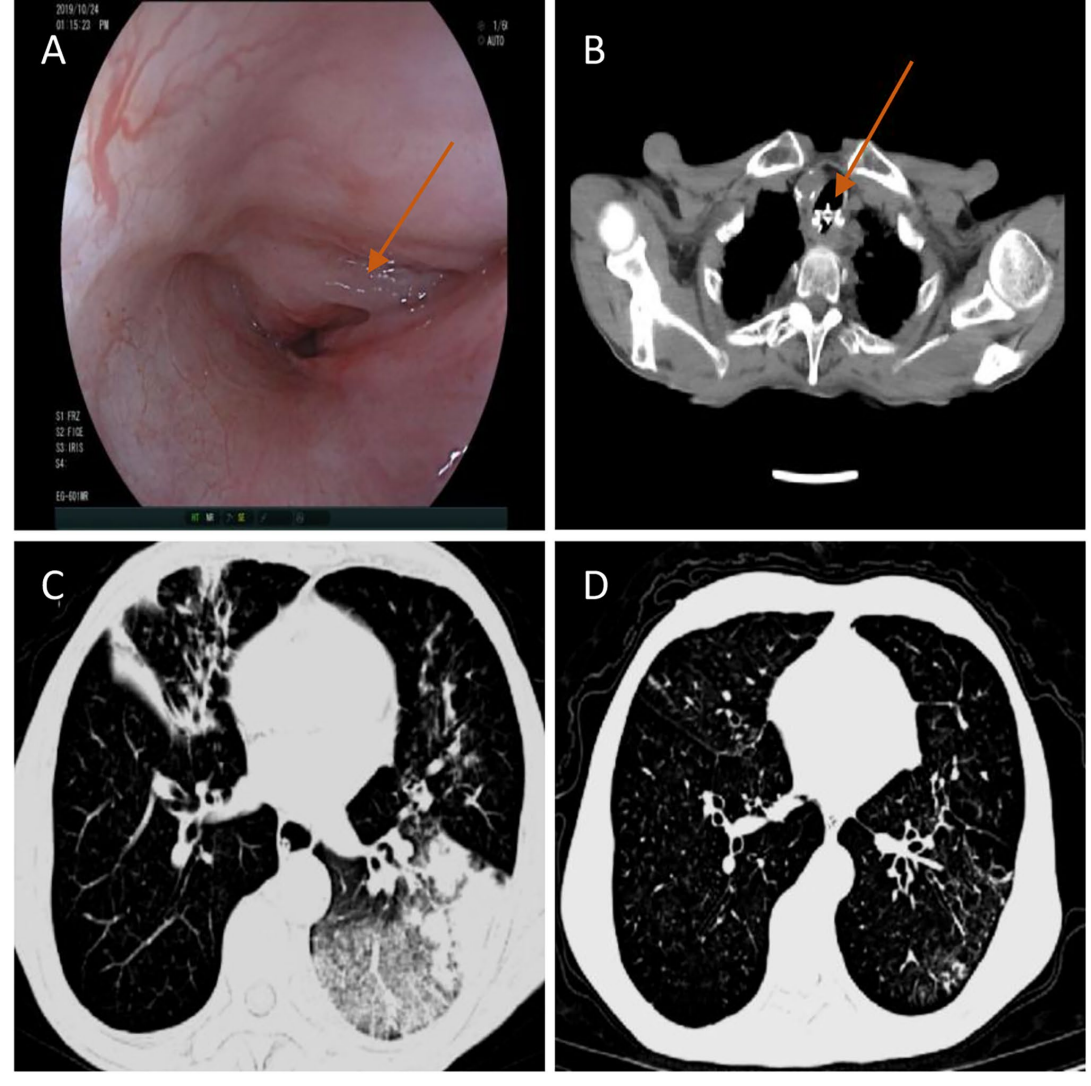
Follow-up evaluation 90 days after TEF occlusion in patients with esophageal cancer. **(A)** Gastroscopy examination showed epithelialization on the surface of the occluder, good adhesion to the esophageal wall, and no residual fistula; **(B)** After 90 days of TEF occlusion, chest CT was performed to confirm the position of the occluder. **(C and D)** Comparison of pre- and postoperative chest CT showed a significant improvement in pulmonary infection after TEF occlusion. The location of the occlusion device is indicated by the arrow

Long-term follow-up includes pulmonary CT, bronchoscopy, or digestive endoscopy to further assess the effectiveness of the occlusion, the status of the occluder endothelization, and the presence of pulmonary infections [Fig. 5]. the three-month postoperative follow-up, bronchoscopy examinations were conducted on all patients, revealing epithelialization of the occluder surface. Among the 8 patients included in the study, 6 patients (75%) exhibited complete endothelialization of the occluder after the closure procedure, while 2 patients (25%) showed partial endothelialization. No visible fistula openings were observed in either the esophageal or airway sides. Notably, there were no instances of coughing or aspiration, and a significant improvement was observed in pulmonary infections. Patients were able to eat without difficulties, and their overall quality of life showed remarkable improvement compared to the preoperative period. Importantly, all patients survived for a period exceeding 3 months (Table 2).

**Table 2:** Selection of the occlude and post-operative follow up at 3-months

Patient number	occluder size(mm)	Endothelialization of the occluder	Sepsis Or lung infection	Complications	Nasal Feeding	Weight(kg)	Survival
1	VSD(9)	completely	improve	None	NO	58	Yes
2	VSD(13)	completely	improve	None	NO	50	Yes
3	ASD(16)	partial	improve	None	NO	70	Yes
4	VSD(12)	completely	improve	None	NO	59	Yes
5	VSD(9)	completely	improve	None	NO	62	Yes
6	VSD(11)	completely	improve	None	NO	65	Yes
7	VSD(13)	completely	improve	None	NO	54	Yes
8	ASD(14)	partial	improve	None	NO	66	Yes

## Discussion

TEF is a rare but life-threatening condition characterized by an abnormal communication between the trachea and esophagus, with an incidence of approximately 1 in 3,500 live births [13, 14]. In adults, TEF can occur as a result of trauma, malignancy, or iatrogenic injury. It is commonly observed in critically ill patients who require prolonged mechanical ventilation and are deemed unsuitable candidates for surgical intervention due to their compromised health status [4, 15]. The majority of TEF cases are located at the mid-tracheal level, predominantly on the posterior tracheal wall, and often present with respiratory infections caused by recurrent aspirations, sepsis, excessive secretions, coughing, and air leakage around the cuffed tube [16]. Spontaneous healing of TEF is rare, and surgical closure is typically required. Surgical repair has been the conventional treatment approach for TEF, involving the closure of the fistula through thoracotomy or laparotomy procedures [1, 17]. The optimal timing for surgery is after the patient has been successfully weaned off mechanical ventilation, the infection has been controlled, and optimal dietary conditions have been achieved [18, 19]. Despite its efficacy, surgical interventions are invasive and associated with a substantial risk of complications, including wound infection, pneumonia, and respiratory distress, especially for the mTEF [4, 20].

Various minimally invasive surgical approaches have been explored for the treatment of TEF. Endoscopic closure techniques involve the use of clips, sutures, or tissue adhesives to directly visualize and close the fistula under endoscopic guidance [21, 22]. Stent placement techniques entail the insertion of a self-expanding metal or silicone tube into the trachea or esophagus to maintain airway or esophageal patency [23–26]. Laser-assisted repair involves the controlled application of laser energy to create a burn and seal the fistula [27, 28]. Despite these minimally invasive techniques offer advantages such as reduced invasiveness and potentially faster recovery, their applicability and efficacy may vary depending on the individual case of TEF. The efficacy of bronchoscopic treatments using substances like fibrin glue, tissue adhesive, and sclerosing agents is limited when the size of the fistula exceeds 3 mm. Airway stents, including silicone stents and self-expandable metallic stents, have demonstrated some effectiveness [29]. However, due to the significant variation in tracheal diameter, stents may not adhere well to the tracheal mucosa, leading to incomplete fistula closure, stent dislocation, and the potential development of fatal tracheal stenosis [30–32]. The success rates of these approaches depend on factors such as the location, size, and nature of the fistula, as well as the overall health status and comorbidities of the patient. Consequently, careful consideration of the individual case is crucial when selecting the most appropriate treatment option especially in mTEF.

Recent studies have reported successful utilization of the cardiac septal occluder for TEF closure, particularly in patients who are not suitable candidates for traditional surgical approaches [12, 33, 34]. The cardiac septal occluder is a self-expanding, double-disc structure composed of tightly woven superelastic nickel-titanium alloy wire. It is primarily designed for closing cardiac septal defects such as atrial or ventricular septal defects. The occluder consists of two discs connected by a waist, with the diameters of the far and near discs being larger than the waist’s diameter. The edges of the discs are slightly concave, allowing them to interlock upon deployment, thereby enhancing the sealing effect. Additionally, a flow-blocking membrane is incorporated within the mesh structure of the occluder to reinforce the sealing capability[Figure 1C] [35].Its potential as a minimally invasive option for TEF treatment has garnered increasing attention. However, there is currently limited evidence regarding the application of cardiac septal occluders for the treatment of mTEF. Therefore, we conducted a retrospective analysis of 8 cases involving patients with mTEF who underwent closure surgery using atrial/ventricular septal defect (ASD/VSD) septal occluder at the Respiratory Department of HuBei Yichang Central People’s Hospital from 2021 to 2023.

In our study, stringent criteria were applied to select mTEF patients for occlusion surgery, taking into account their severe conditions such as advanced malignant tumors and overall poor health, which made conventional surgical intervention inappropriate. Additionally, comprehensive preoperative examinations played a pivotal role in patient selection. These adjunctive diagnostic procedures commonly employed in mTEF diagnosis not only facilitated prompt and accurate localization, sizing, and etiological identification of the fistula but also facilitated postoperative follow-up after occlusion therapy. Chest CT, bronchoscopy, and digestive endoscopy were the most frequently utilized adjunctive diagnostic procedures for TEF diagnosis. Successful execution of the occlusion surgery also necessitated collaborative efforts among respiratory physicians, cardiologists, gastroenterologists, anesthesiologists, and operating nurses.

Based on our experience in treating 8 cases of mTEF, we propose the use of an occluder waist diameter that is 3–4 mm larger than the length or diameter of the fistula to ensure complete coverage by the double discs. In terms of selecting ASD or VSD occluders for TEF closure, our study provides the following recommendations: for TEFs with a larger fistula diameter and larger esophageal and tracheal openings (> 1 cm), an ASD occluder is recommended. Conversely, for TEF with a smaller fistula diameter and smaller esophageal and tracheal openings (< 1 cm), a VSD occluder is preferred. VSD occluders are smaller and have a narrower waist compared to ASD occluders. The appropriate selection of occluders is crucial as an oversized occluder may compromise tissue circulation, leading to tissue damage and potential complications, while an undersized occluder may result in displacement or detachment. The complications associated with cardiac septal occluder treatment for malignant tracheoesophageal fistula encompass occluder displacement, tissue damage, infection, re-intervention, and persistent symptoms such as dysphagia and dyspnea. Occluder displacement poses a risk of inadequate closure, potentially leading to symptom recurrence, while insertion and deployment of the device may cause tissue damage, inflammation, or perforation. The presence of the occluder increases the risk of infection, necessitating antibiotic therapy. In cases of complications, re-intervention may be required to address issues such as occluder migration or inadequate closure, prolonging the recovery period and increasing the risk of further complications. Persistent symptoms such as difficulty swallowing or breathing may persist due to inadequate closure or occluder-related issues, impacting the patient’s quality of life and requiring additional management strategies.

Postoperative follow-up assessments revealed that the utilization of a cardiac septal occluder is a safe and effective approach for mTEF closure. Despite the three-month follow-up, two patients still exhibited partial endothelialization of the occluder. This is primarily attributed to the use of larger cardiac septal occluders due to the presence of a large mTEF. However, it is noteworthy that there was a significant improvement in pulmonary infections, allowing patients to eat without difficulty. Moreover, there was a remarkable enhancement in their overall quality of life compared to the preoperative period. Furthermore, no major complications were observed, and patients did not experience choking or aspiration following mTEF closure surgery. The utilization of the cardiac septal occluder offers several advantages, including shorter hospital stays and faster recovery times compared to traditional surgical techniques, with fewer postoperative complications such as wound infections and scarring. Importantly, the survival period exceeded the median survival period of 6 to 12 weeks.

## Conclusions

In conclusion, that the use of a cardiac septal occluder could potentially emerge as a minimally invasive palliative alternative for treating mTEF. This approach shows promise as a treatment option for TEF closure, with high success rates and minimal complications. However, large-scale studies are needed to evaluate the long-term outcomes and safety of this technique [36]. Further clinical studies are warranted to establish standardized protocols, assess long-term outcomes, and compare the effectiveness and safety of the cardiac septal occluder with other minimally invasive techniques and traditional surgical approaches.

### Electronic supplementary material

Below is the link to the electronic supplementary material.

Supplementary Material 1

## Data Availability

No datasets were generated or analysed during the current study.
